# Symptomatic Gallstones in Young Patients Under the Age of 30 Years

**DOI:** 10.7759/cureus.19894

**Published:** 2021-11-25

**Authors:** Asif Ali, Sughra Perveen, Imran Khan, Tanweer Ahmed, Ali Nawaz, Abdul Rab

**Affiliations:** 1 Department of Surgery, Jinnah Postgraduate Medical Centre, Karachi, PAK; 2 Department of General Surgery, Jinnah Postgraduate Medical Centre, Karachi, PAK

**Keywords:** gallbladder, cholelithiasis, young patients, symptomatology, diabetes mellitus, obesity

## Abstract

Introduction

Gallstones are deposits of digestive fluid that is hardened into small pellets. Gallstones can be symptomatic or asymptomatic. The present study assessed the sociodemographic and clinical characteristics of patients under the age of 30 years old with symptomatic gallstones.

Methodology

A prospective, observational study was conducted at Surgical Unit-3, at Jinnah Postgraduate Medical Centre (JPMC), Pakistan, from July 2020 to July 2021. All patients suspected of having gallstone disease underwent ultrasonic examination. A diagnosis of gallstone disease was made if the gallbladder showed a hyperechoic mass casting a posterior acoustic shadow, with a change of position. All of the patients with gallstone disease were hospitalized. The patients were asked about their age, past history (with special emphasis on last pregnancy and years of oral contraceptive use, if any), socioeconomic status, and other demographic data. The patients were treated with a cholecystectomy, either laparoscopic or open. Postcholecystectomy complications, if any, were recorded.

Results

A total of 210 patients under the age of 30 years were included. The mean age of patients was 25 ± 3 years. The majority of the population were females, i.e., 170 (81%). Upon assessing the risk factors, it was found that 31.84% of the female patients had used oral contraceptives, 32.96% were pregnant at the time of presentation, 20.48% had diabetes mellitus, and 27.14% had a history of gallstones. Furthermore, it was found that about 114 (54.29%) patients were overweight with a body mass index (BMI) of 25-30 kg/m^2^. High triglyceride levels and high cholesterol levels were found in 108 (51.43%) and 115 (54.76%) of the patients, respectively, indicating a link between symptomatic gallstones and younger age.

Conclusion

Our study indicated that young people are prone to symptomatic gallstones. The rate of disease was considerably high among females and in patients with high cholesterol and triglyceride levels and abnormal body mass index. Further exploratory studies are needed to determine the cause of gallstones.

## Introduction

Gallstones are deposits of digestive fluid that is hardened into small pellets. Gallstones can be symptomatic or asymptomatic [1]. The size of gallstones varies from grains of sand to golf balls. Gallstone attacks usually cause vomiting, nausea, and pain in the stomach, ribs, and between the patients’ shoulders. In some severe cases, it may cause complications such as acute pancreatitis, jaundice, bowel obstruction, and gallbladder carcinoma and may require cholecystectomy [2,3].

Gallstone is a very common disease, and it has been classified into two types: cholesterol stone, which has >70% cholesterol content, and pigment stone, which has <30% cholesterol content [4]. A study on postoperative patients revealed that the rate of symptomatic cholelithiasis was 3.5%, with a mean duration of symptoms of around 12 months. They revealed that sudden weight loss was a significant factor that led to the development of cholelithiasis [5]. Females have a higher chance to develop cholelithiasis. This may be due to hormonal and reproductive factors, i.e., oral contraceptives and pregnancy. Another study from India revealed that the incidence of cholelithiasis is 21.76% among patients who underwent a bariatric procedure [6].

Literature showed that cholelithiasis is strongly related to body mass index (BMI) [7,8]. This may be due to increased fat consumption and less physical activity. The prevalence of gallstones is also very common in the upper and middle socioeconomic class due to their dietary habits and obesity. The treatment of gallstone depends upon how many symptoms a patient is showing [9,10]. For symptomatic gallstones, surgery is performed to remove gallstones from the gallbladder. However, due to the scarcity of local literature, we cannot ascertain the epidemiology of gallstones in the younger Pakistani population. Thus, the current study was conducted to find the sociodemographic and clinical characteristics of young patients with symptomatic gallstones.

## Materials and methods

From July 2020 to July 2021, a prospective, descriptive study was conducted at Surgical Unit-3, at Jinnah Postgraduate Medical Centre (JPMC). The ethical committee of JPMC granted approval for this study with the reference number F9-98-IRB-2018-G/28867/JPMC. A total of 210 patients under the age of 30 years, irrespective of gender, with a diagnosed gallstone disease were included in the study group. Informed written consent was obtained from all patients. All those who presented with the symptomatology of biliary colic without any evidence of gallstones were excluded.

All patients suspected of having gallstone disease underwent ultrasonic examination. A fasting period of 12 hours was required prior to the examination, which was carried out in the supine position. A diagnosis of gallstone disease was made if the gallbladder showed a hyperechoic mass casting a posterior acoustic shadow, with a change of position. All of the patients with gallstone disease were hospitalized. Each participant was personally interviewed by trained interviewers.

The patients were asked about their age, past history (with special emphasis on last pregnancy and years of oral contraceptive use, if any), socioeconomic status, and other demographic data. Family histories of gallstones and diabetes mellitus were also determined. Basic data on weight with the patient clothed and height without shoes was documented. Body mass index (kg/m^2^) was calculated as a measure of obesity. The patients were categorized as normal weight when their BMI is less than 25, overweight when their BMI is 25-30, and obese when their BMI exceeds more than 30.

All patients underwent laboratory analysis for a complete blood picture with reticulocyte count, serum transaminases, bilirubin level, and fasting lipid profile. The patients were treated with a cholecystectomy, either laparoscopic or open. Postcholecystectomy complications, if any, were recorded. Data were recorded and analyzed using the Statistical Package for the Social Sciences (SPSS) version 16.0. Descriptive statistics were calculated for both qualitative and quantitative variables. Mean ± SD was calculated for demographic data.

## Results

A total of 210 patients under the age of 30 years were included. The mean age of the patients was 24.65 ± 3.25 years. The majority of the population were females, i.e., 170 (80.9%) (Table 1).

**Table 1:** Characteristics of the Study Population

Characteristics	n (%)
Gender	
Male	40 (19.04%)
Female	170 (80.9%)
Age	24.65 ± 3.254
Socioeconomic status	
Lower	33 (15.71%)
Middle	136 (64.76%)
Upper	41 (19.52%)

The most frequent symptom the patient presented with was biliary colic (n = 59, 28.1%), followed by chronic cholecystitis (n = 46, 21.9%) and then acute cholecystitis (n = 38, 18.1%) (Table 2).

**Table 2:** Symptoms Presented by the Study Population

Symptoms	n (%)
Biliary colic	59 (28.1%)
Acute cholecystitis	38 (18.1%)
Chronic cholecystitis	46 (21.9%)
Acute chronic cholecystitis	26 (12.38%)
Acute pancreatitis	34 (16.19%)
Choledocholithiasis	7 (3.33%)
Cholecystoenteric fistula	0 (0%)
Biliary ileus	0 (0%)

Upon assessing the risk factors, 31.84% of the patients had used oral contraceptives, 32.96% were pregnant, 20.48% had diabetes mellitus, and 27.14% had a history of gallstones. Furthermore, about 114 (54.29%) patients were overweight, with a body mass index of 25-30 kg/m^2^. High triglyceride levels and high cholesterol levels were found in the majority of our study population, which indicated a link between symptomatic gallstones and younger age (Table 3).

**Table 3:** Distribution of Risk Factors Associated With the Incidence of Gallstones Among the Participants

Gallstones	
Risk factor	n (%)
Oral contraceptive usage	
Yes	57 (31.84%)
No	122 (68.16%)
Pregnancy	
Yes	59 (32.96%)
No	120 (67.04%)
Diabetes mellitus	
Yes	43 (20.48%)
No	167 (79.52%)
History of gallstones	
Yes	57 (27.14%)
No	153 (72.86%)
Socioeconomic status	
Lower	33 (15.71%)
Middle	136 (64.76%)
Upper	41 (19.52%)
Body mass index (kg/m^2^)	
<25 (normal)	74 (35.24%)
25–30 (overweight)	114 (54.29%)
>30 (obese)	22 (10.48%)
Cholesterol level (mg/dL)	
<200	95 (45.24%)
>200	115 (54.76%)
Triglyceride level (mg/dL)	
<150	102 (48.57%)
>150	108 (51.43%)

## Discussion

In our study, we found that young people (mean age: 24.65 ± 3.25 years) are more prone to symptomatic gallstones. Gallstones are the most common pathology of the hepatobiliary system with a high prevalence rate among females (80.9%) as compared with males (19.04%). Females were more commonly seen with this disease due to a number of factors such as oral contraceptive use and pregnancy. The incidence of this disease in young individuals has increased so rapidly, which in turn costs a lot to the healthcare system, especially in a third-world country such as Pakistan.

A similar study to ours was conducted by Shafique et al., in which 48.13% of the patients were from an age group below 30 years of age [11]. The study discussed factors such as high body mass index (BMI) and dyslipidemia to be important prognostic factors for gallstones. This was similar to our study, in which 114 (54.29%) of the population was overweight, and cholesterol levels greater than 200 mg/dL were seen in 115 (54.76%) of the participants. Constantinescu et al. identified patients between the ages of 16 and 25 and found obesity, pregnancy, age, and female gender as important risk factors for the development of gallstones for this age group [12]. The authors also identified that the most commonly seen complications of the disease in young individuals were acute pancreatitis and the presence of stones in the duct. Similarly, Kim et al. studied the young population of Korea, where causes of obesity, such as high BMI, cholesterol, HDL levels, LDL levels, and size of the waist, were risk factors for the development of disease in the young population [13]. These findings were similar to other studies [14,15].

Another study by Sun et al. found that middle- to high-income class individuals was more at risk for developing gallstones [16]. In the present study, individuals belonging to the middle class were more prone to developing gallstones. Sun et al., however, found old age to be a significant contributor to the development of gallstones, not in adolescents below 19 years of age. The elderly were commonly seen with gallstone disease; this was attributed to the aging and sedentary lifestyle of the elderly population. Similarly, Mora-Guzmán et al. revealed that elderly patients were at a higher risk of developing gallstones and gallstone-related complications along with recurrence of gallstones in one-third of patient follow-ups [17].

Overall, our study coincided very well with the current literature. Nevertheless, the study had some limitations. For instance, a larger sample size would have permitted more insight into the factors leading to gallstone formation in the young populations.

## Conclusions

Our study indicated that young people are prone to hepatobiliary issues, with gallstones being the most frequent. The rate of disease was considerably high among females and in patients with high cholesterol and triglyceride levels and abnormal body mass index. Further exploratory studies are needed to determine the cause of gallstones.
